# Notes and News

**Published:** 1902-08-02

**Authors:** 


### NOTES AND NEWS.

THROUGHOUT the week small-pox cases in the metropolis has shown a steady decrease.

MRS. SAMUEL WALLER has sent her quarterly gift of £2,500 to King Edward's Hospital Fund for London.

MR. LUCAS TOOTH has placed £10,000 at the disposal of the King, who has presented it to his Hospital Fund.

AN automobile ambulance is to be constructed for the Ambulance Department of the Metropolitan Asylums Board.

NINETY-FIVE cases of cholera have been reported at one time in Cairo this week. At Moucha cases have been continuously reported and the mortality throughout the epidemic has been high.

THE old Physic Garden at Chelsea, once the property of the Apothecaries' Society, has been resuscitated, and was opened in its new garb on Friday last by Lord Cadogan, in the presence of a learned and distinguished gathering. The Garden will serve as a studying ground for various teaching centres, and complete laboratories and greenhouses have been prepared for the purpose. The Garden is on the Embankment, and is surrounded by modern houses. Formerly the river encroached into the grounds.

WASHINGTON is to have a large new municipal hospital which will contain the novel combination of accommodation for general cases, tuberculosis, and infectious diseases. The hospital will be divided into three groups and consist of 37 buildings. The general hospital will occupy 14 two-storey ward buildings, the tuberculosis division, two large and one small ward buildings, and the contagious division will consist of four large-ward buildings. Each division is to have separate administration accommodation, with central lighting and heating plant to serve the whole institution. It is intended to provide for 1,150 patients. The architects are Messrs. Day, of Philadelphia. The hospital will stand on a site of 32 acres, 2¼ miles from White House.

LORD AND LADY LEICESTER have given £1,000 towards establishing a consumption sanatorium for Norfolk. A site of forty-five acres, and a house which will be remodelled and enlarged, have been bought, and have been presented to the Committee by a gentleman who will further help the scheme by endowment.

THE Cancer Research Fund will commence operations before very long. There is now £36,500 to the credit of the Fund, of the £100,000 deemed necessary. The Prince of Wales will become President, and the Executive Committee includes Sir William Broadbent, Sir William Church, and Professor Sims Woodhead.

THE President of the Suez Canal Company, Prince Auguste d'Arenberg, has asked for the co-operation of the Liverpool School of Tropical Medicine to cope with malaria at Ismailia, and the services of Major Ronald Ross are especially requested in the matter of dealing with the mosquitos. The School will accede to the request and it is hoped that Major Ross will proceed to Ismailia in September.

THE plans of Messrs. J. Simpson and E. Milner Allen for the New Manchester Royal Infirmary show accommodation for 452 beds. Twenty beds will be reserved for Jewish patients, and accommodation provided for 230 officers, nurses and servants. There are to be five operating theatres and clinical and bacteriological laboratories. The cost is to be about £200,000.

THE medical officer of the London County Council makes a report which is encouraging to dwellers in London. He states that the expectation of life in London is greater now than it was twenty years ago. He will improve on his statement probably when the automobile has usurped horse traction, and the insanitary and crowded areas are gradually swept away.

WE note that in the *Morning Leader* there is a letter from the Hon. Stephen Coleridge, in the course of which he writes as follows in reference to the article on the Hospital of St. Francis which recently appeared in our columns :—"In the first place let me say that I fully endorse the general criticisms of the inadequacy of the building, both as regards space and general fitness, for the work that is attempted to be done in it."

THE *Voyage d'Etudes Medicales* bids fair to become a permanent institution. This year it is proposed to visit the baths, climatic health resorts, etc., in the eastern districts of France and the Vosges. Reduced railway fares are offered in France to the station Vittel, at which the tour commences, and from Besançon, where it ends. For the tour itself, from the moment of arrival at Vittel (September 7th) to the time when the travellers separate at Besançon (on the evening of September 16th or the morning of September 17th), the price is 200 francs, payable in advance—before August 25th. This includes railway and carriage fares, hotels, refreshments, transport of luggage, and *pourboires.* For further information application should be made to Docteur Carron de la Carrière, 2 Rue Lincoln, Paris.

